# Guest edited collection: fungal evolution and diversity

**DOI:** 10.1038/s41598-023-48471-0

**Published:** 2023-12-05

**Authors:** Robert Russell Monteith Paterson, Zakaria Solaiman, Oscar Santamaria

**Affiliations:** 1 Centre for Biological Engineering, Gualtar Campus, University of Minho, 4710 057 Braga, Portugal; 2 The UWA Institute of Agriculture and UWA School of Agriculture and Environment, The University of Western Australia, Perth, WA 6009 Australia; 3 Department of Plant Production and Forest Resources, Sustainable Forest Management Research Institute, Higher Technical School of Agricultural Engineering, Universidad de Valladolid, Avda. Madrid 57, 34004 Palencia, Spain

**Keywords:** Evolutionary ecology, Fungal evolution, Fungal ecology

## Abstract

There are 5 million fungal species. However, the discovery and classification of fungi are in high flux. Modern concepts indicate that the three kingdoms of “fungi” are *Chromista, Fungi* and *Protozoa*. Strong support for the wrong phylogeny can occur without correct analytical methods. In the current Collection we envisaged fungi representing extremely diverse and ancient eukaryotic organisms, with familiar groups such as mushrooms, yeasts and “moulds”. We collected 6 fascinating papers in three areas of Diversity, Chemical Diversity and Evolution.

The practical definition of “fungi” being organisms studied by mycologists” ^1^ is tautological. In addition, some scientists who study fungi may not primarily be mycologists. Fungi were considered until recently to form a single kingdom separate from, for example, animals, bacteria, plants, and protists. Modern concepts indicate the three kingdoms of “fungi” are *Chromista, Fungi* and *Protozoa* and, almost counter-intuitively, *Fungi* are more closely related to animals than plants by nucleic acid (NA) analysis. They were studied by botanists perhaps because fungi are often associated with plants. Initial indications are that *Animalia* and *Fungi* are sister groups which arose from a protozoan ancestor similar to a chroanozoan and fungi diverged from the animals ca. 1.3 billion years ago^2^. No traits are shared by all fungi to define the kingdom, although they are characterized usually by chitinous cell walls and employ osmotrophy. The presence of ergosterol was considered indicative of an organism being fungal, however, Richards et al. ^3^ stated that there is no synapomorphy for the fungi and we should simply “get used to it”. The discovery and classification of fungi are still in high flux particularly among the more basal branches of the tree. NA systematics has been embraced and true diversity revealed from genomic and environmental surveys^2^. Isolating, preserving and culturing fungi can cause mutational pressure and/or physiological change^4^ and the environmental, uncultured fungi may be more representative of wild type taxa in some cases. Individual cultures or specimens for NA systematic analysis should be obtained from three collections/biological resource centres to combat potential variation^5^. The creation of innovative developments such as the Microbial Resource Research Infrastructure^6^ will greatly facilitate this requirement.

Species delineation is optimal when a multi-disciplinary approach is taken in the selection of characters, often including morphological, NA, other biochemical and physiological analyses^5^. However, the cryptic and microscopic nature of many fungi means diversity is under-sampled and less than 5% of the estimated 5 million species have been described formally. There are many fungi discovered by analysing environmental samples using NA methods, i.e. the dark matter taxa and uncultured majority^2^: dark matter taxa will increase the number greatly.

A comprehensive phylogeny has not yet been constructed and there are questions that need addressing^2,5^. The huge volume of data from the genomic revolution is a challenge. Strong support for the wrong phylogeny can occur without careful consideration of analytical methods and other analytical challenges are gene duplications, horizontal gene transfer and population-level processes to determine true species phylogeny. Finally, how can unculturable taxa be included in the growing tree^2^?

We envisaged fungi as extremely diverse and ancient eukaryotic organisms, represented by familiar forms such as mushrooms, yeasts and “moulds” in this Collection of papers. Species are distributed over the globe, the atmosphere and almost all habitats. Fungi are key players in ecological communities, often forming symbiotic relationships with other species and are vital decomposers and nutrient recyclers. The economic value of fungi is immense and demonstrates their utility more generally when they are exploited as food, pharmaceuticals and other industrial products. The Collection welcomed research in the fields of conservation, ecology, evolution and chemical diversity.

## The collection

The collection included six papers in three broad areas: Diversity, Chemical Diversity and Ecology. A hugely important paper is Franic et al.^7^ who analysed records of insects and fungi collected from dormant twigs from 155 tree species at 51 botanical gardens/arboreta in 32 countries. The increasing importance of high temperatures on differences in studied communities indicate that climate change could affect tree-associated organisms directly and indirectly through host range shifts. The results of this study highlight the need to limit the establishment of tree pests and increase the resilience of forest ecosystems to changes in climate. In another major study, Sanz-Benito et al.^8^ revealed that *Quercus* stand types are richer in fungal diversity than *Cistus* types, particularly for the more mature stands. Furthermore, ectomycorrhizal (ECM) diversity demonstrated a similar trend, with an increase in short-distance exploration fungi with age, whereas long-distance exploration fungi did not vary. The greatest overlap in terms of ECM fungal community composition during the successional process occurred between old *Cistus* understory and young *Quercus* trees by compositional and network analyses. *Inocybe, Amanita, Lactarius, Russula, Tomentella* and *Cortinarius* played a key role as bridging species between the two host species. These require more intense study to understand their role as *Cistus* fields are replaced by oak forests and their possible engagement in tree recruitment and/or seedling survival.

In one of the most inhospitable places on earth, the Antarctic, fungi may survive by producing novel secondary metabolites, for example, to outcompete microorganisms (e.g. other fungi, bacteria and protozoa), due to the toxicities of the compounds. The new metabolites could be much needed antibiotics to combat drug resistance. Related to this possibility, four fungi were isolated from the region by Ordóñez-Enireb et al.^9^ which were identified as *Antarctomyces* sp., *Thelebolus* sp., *Penicillium* sp., and *Cryptococcus gilvescens.* They were shown to have varying degrees of antibacterial activity and indicate clearly the need to preserve the Antarctic as a potential source of useful fungi. The compounds responsible for the activities require identification and species names will be essential in all cases in future work.

Kakizaki et al.^10^ is one of two papers concerning chemical diversity per se. The important model fungus *Coprinopsis cinerea* was used to report on septins, regulators, the nuclei and histone H1, used to examine differentiation in the formation of fruiting bodies. These base line data can be compared to the situation in other specimens or taxa. *Sclerotinia sclerotiorum* causes stem rot in plants and is one of the major pathogens of oilseed *Brassica*^11^. The investigation offers a better understanding of the *S. sclerotiorum* genome, secretome, and its effector repertoire, crucially refining the present knowledge on *S. sclerotiorum*–*Brassica* interactions and the necrotrophic lifestyle of the phytopathogen in general.

Christinaki et al.^12^ represent the evolutionary aspects of the present Collection. The authors discuss co-evolution of large, inverted repeats (LIRs) and G-quadruplex (G4) DNA elements in fungal mitochondria which may facilitate mitogenome stability. Their fungus of choice was *Malassezia.* Members of this basidiomycetous yeast are important components of the human skin microbiome, and associated with bloodstream infections, various skin diseases, gut diseases and certain cancers. Large LIRs and G4 DNA elements coexist and evolved convergently providing genome stability through recombination. The mechanism is common in chloroplasts but rarely found in mitogenomes.

Species concepts for fungi are indeed challenging in many cases and universal characters remain elusive. The genomic revolution is a challenge and a “blessing” where a polyphasic approach to identification is considered optimal^5^. Robust evidence for an inaccurate phylogeny is a concern unless the analytical methods employed are understood. To reiterate, horizontal gene transfer, gene duplications and population-level processes require consideration when assessing true species phylogeny and unculturable taxa require inclusion. We are convinced the quality and importance of the new research in this Collection makes a highly significant contribution to the fields.

In conclusion, we see here how the diversity of fungi encompass a large range of fascinating and potentially valuable topics. The evolution of the fungi is much more complex than was previously thought. Diversity is almost too large to comprehend, especially when dark matter taxa are considered and much more work is required on the taxonomy and nomenclature of these organisms. There is so much more to be discovered especially when considering the small number of species described compared to the large total. More bioprospecting projects are required to isolate fungi and identify the bioactive metabolites they produce from unexplored environments such as rainforests and the Antarctic^9,13^. This is indeed big science! It would be remiss not to mention further the very worrying implications of climate change, better described as the climate emergency. Just how many taxa that may become extinct could form the basis of a concept paper, equivalent to those that estimate the total number of fungal species^2,3,5^. Surely it is a minimum requirement that net zero is met by 2050? as promised by many governments and to save our precious fungi.
